# Low ovarian responders produce more progesterone per follicle than normal and high responders

**DOI:** 10.5935/1518-0557.20240043

**Published:** 2024

**Authors:** Alfredo Cortés-Vazquez, Alfredo Leonardo Cortés-Algara, Daniel Moreno-García, Johnny S. Younis

**Affiliations:** 1 Reproductive Endocrinology Department, Centro Médico Nacional 20 de Noviembre, Mexico City, Mexico; 2 Minimally Invasive Gynecology and Robotic Surgery Department, Centro Médico Nacional 20 de Noviembre, Mexico City, Mexico; 3 Azrieli Faculty of Medicine, Bar-Ilan University, Safed, Israel

**Keywords:** progesterone, low responders, index, follicle

## Abstract

**Objective:**

Late follicular premature progesterone rise is a complex phenomenon encountered during assisted reproductive technology (ART) treatments; different etiologies can occur in the same patient. Low ovarian responders may be the best example, since higher FSH doses and ovarian aging-related changes may interact and generate a premature progesterone rise. This study aims to explore the correlation between progesterone levels on hCG day and the progesterone-to-follicle index and compare the progesterone-to-follicle index according to ovarian response.

**Methods:**

We performed a retrospective, observational, analytic, cross-sectional, and cohort study at the Reproductive Endocrinology Department at Centro Médico Nacional 20 de November between January 2015 to January 2020. After verifying for normalcy, a Spearman Rho, Principal Component Analysis, and a simple linear regression model were performed. Treatment cycles were classified according to their ovarian response. Low-ovarian responders were classified according to the Bologna Criteria. Then an ANOVA test was performed to compare each group.

**Results:**

Our results show that the progesterone-to-follicle index correlates best with progesterone levels on hCG day. Comparing all the ovarian responses, low ovarian responders have the highest progesterone-to-follicle index of the four groups.

**Conclusions:**

Low ovarian responders produce more progesterone per follicle than regular and high responders.

## INTRODUCTION

Premature progesterone elevation in the late follicular phase is an intricate phenomenon during controlled ovarian stimulation (COS) for IVF/ICSI treatments. Until recently, premature progesterone elevation in stimulated cycles employing gonadotropin releasing-hormone (GnRH) analogues was misleadingly described as “premature luteinization” (Fatemi & Van Vaerenbergh, 2015). This phenomenon has been shown to occur in 20.8 to 38.3% of all IVF/ICSI cycles, irrespective of the COS protocol employed (Bosch *et al*., 2003; Ubaldi *et al*., 1996). Some authors believe premature progesterone elevation may have different etiologies in accordance with the ovarian response; in high responders, multiple ovarian developments may cause progesterone accumulation, while in low responders, follicular aging may lead to progesterone elevation in contrasted mechanisms (Cortés-Vazquez *et al*., 2022). Nevertheless, this concept was not further explored in the literature.

Since 2001 several authors have addressed that premature progesterone elevation could be an early sign of low ovarian reserve (Younis *et al*., 2001). In 2019 a novel mechanism was proposed by which low ovarian reserve manifested by follicular senescence could lead to premature progesterone elevation (Younis, 2019). Delicate paracrine and autocrine mechanisms within the oocyte were suggested to preserve preovulatory follicle integrity, which may be disrupted in cases with low ovarian reserve. In 2015, a novel measure was introduced to evaluate intrinsic follicular development in relation to follicular response. The progesterone-to-follicle index was calculated by dividing the late follicular serum progesterone by the number of follicles ≥14 mm (Shufaro *et al*., 2015). Late follicular progesterone increase was shown to be detrimental to clinical pregnancy rate if it is a consequence of increased progesterone production per follicle (high progesterone-to-follicle index) but not if it is a consequence of additional follicular recruitment. However, this measure was not further investigated in the clinical setting.

Our study aims to explore late follicular progesterone elevation in accordance with ovarian response in the IVF setting. The study aims to explore the correlation between progesterone-to-follicle index and serum progesterone levels on human chorionic gonadotropin (hCG) day and compare the progesterone-to-follicle index in accordance with the ovarian response.

## MATERIAL AND METHODS

### Study population and study design

This retrospective, observational, analytic, cross-sectional, and cohort study was performed at the Reproductive Endocrinology Department of the Centro Médico Nacional 20 de Noviembre in Mexico City between January 2015 to January 2020. All couples underwent basic infertility tests, including day-3 antral follicle count (AFC), day-3 serum FSH, LH, estradiol, progesterone, prolactin, and semen analyses for the male partner.

All women included in the study received a conventional COS protocol and underwent an IVF/ICSI-ET treatment. Women undergoing natural cycles, double ovarian stimulation, or stimulation with clomiphene citrate were excluded from the evaluation. Furthermore, women with incomplete medical records or oncologic patients undergoing fertility preservation were excluded.

The protocol of the study was approved by the Ethics Committee (Institutional Review Board, reference number 263.2021) and authorized the chart review of 498 infertile couples who underwent 534 cycles. Written informed consent was waived owing to the study’s retrospective nature, and patients’ data were used anonymously.

### Ovarian stimulation protocols

Patients started ovarian stimulation on menstrual cycle day 2 or 3. Gonadotropin dosages’, rFSH (Gonal F, Merck-Serono, Italy) and rLH (Luveris, Merck-Serono, Italy) were determined according to age, body mass index (BMI), and previous ovarian response. The duration and dosage of rFSH varied case-by-case according to the follicular response. A flexible GnRH antagonist treatment (0.25 mg/day, Cetrorelix, Cetrotide, Merck, Geneva, Switzerland) was started when the leading follicle reached 12 to 14 mm. Patients should have at least three leading follicles with a mean diameter beyond 18 mm for final follicular triggering. A 250 mcg recombinant hCG dosage was employed subcutaneously (Ovidrel, Merck-Serono, Italy).

### Follicular follow-up

All patients had transvaginal sonography (TVS) at menstrual cycle day 2 or 3. From day 8 of the cycle, TVS was carried out every two days to monitor follicular development. Experienced doctors carried out all follicular measurements using a Voluson S10 Expert (GE Health Care, Parramatta, Australia) or a Clear Vue 350 (Philips, USA) with a vaginal probe. Concomitantly, serum FSH, LH, estradiol and progesterone levels were determined.

### Oocyte retrieval

Oocytes were retrieved transvaginally under ultrasound guidance 34-36 hours after hCG administration. The oocytes were graded for maturity based on the morphological characteristics, as previously described (Cortés-Vazquez *et al*., 2022).

### Outcome measures

Age, BMI (kg/m2), number of follicles ≥14 mm, serum progesterone (ng/mL) and estradiol (pg/mL) levels on hCG day, number of retrieved oocytes, mature oocytes and oocyte maturation rate were recorded and analyzed.

The primary outcome measures were to explore the correlation between serum progesterone levels and progesterone/follicle index on hCG day. In addition, to compare the progesterone/follicle index between high, normal, suboptimal, and poor ovarian responders. The secondary outcome measure was to define the group of patients with the highest progesterone-to-follicle index after an IVF/ICSI cycle.

### Statistical analysis

A non-probabilistic convenience sampling method was performed. A Spearman Rho analyzed variables that failed to have a normal distribution; otherwise, they were analyzed by a Pearson’s coefficient. Continuous data were verified for normalcy using the Kolmogorov-Smirnov test. After that, simple linear regression and principal component analyses were performed. A method of data reduction named Principal Component Analysis was performed on raw data to make a correlation matrix. Principal Component Analysis was used to outline the patients with the highest and lowest progesterone-to-follicle index groups. All the patients were divided into groups according to their ovarian response and the study design. It is important to point out that only low ovarian responders were defined according to Bologna Criteria. Group 1, low ovarian responders, less than 3 oocytes, Group 2, suboptimal response, 4 to 7 oocytes, Group 3, normal responders with 8 to 14 oocytes and Group 4, high responders, more than 15 oocytes. A two-sided p-value of <0.05 was accepted as statistically significant. Data are presented with the mean and the corresponding standard deviation. Statistical analysis was conducted using the SPSS version 23 (IBM Corp., USA).

## RESULTS

A total of 568 cycles completed the inclusion criteria, and after discarding patients with incomplete records, 534 cycles were eligible for evaluation. At Table 1, we show our patient’s demographic and clinical information. The mean age of our patients is 35.00±3.55years (18 to 44 years), and the mean BMI is 25.80±3.90kg/m2. The Kolmogorov-Smirnov test failed to demonstrate normalcy in our data.

**Table 1:** Patients demographic and clinical information (n=538).

Parameter	Mean	SD
Age (years)	35.76	± 3.55
BMI (kg/m^2^)	25.88	± 3.90
Stimulation length (days)	9.9	± 1.57
FSH Dosage (IU)	2438	± 957
Num. follicles >14 mm	8.78	± 5.27
Progesterone (ng/mL)	1.13	± 1.32
Estradiol (pg/mL)	2667	± 5346
Num. oocytes retrieved	6.24	± 4.68
Progesterone-to-follicle index (pg/follicle)	0.1638	±.1865

BMI= Body Mass Index. SD=standard deviation.

**Figure 1:**
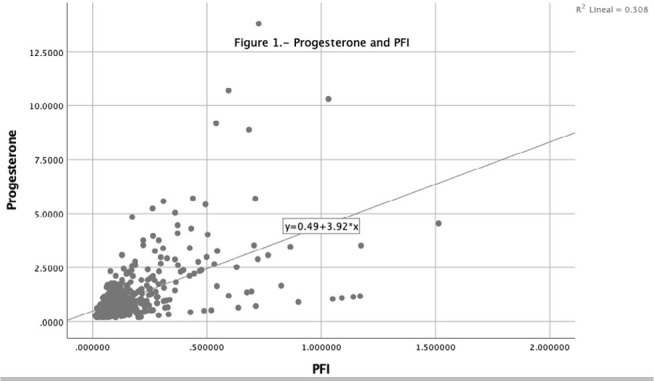
Progesterone and PFI.

The Spearman Rho showed that progesterone on hCG day has a positive correlation with PFI (r=.584), a positive correlation with the number of follicles (>14 mm, r=.355), and a positive correlation with the number of oocytes retrieved (r=.308). All of these correlations are statistically significant and are summarized in Table 2. It is essential to point out that the progesterone-to-follicle index negatively correlates with the number of retrieved oocytes, the number of follicles >14 mm, and the estradiol levels on hCG day (r= -.318, -.477, and -.186, respectively). All of the negative correlations were statistically significant. A simple linear regression model showed a positive trend between progesterone levels on hCG day and progesterone-to-follicle index, with a significant 3.916 Beta Coefficient (Figure 1).

**Table 2:** Correlation coefficient tests of ovarian stimulation parameters and progesterone levels on HCG day.

Parameter	Correlation Coefficient
Num. oocytes retrieved	.308^**^
Estradiol	.348^**^
Num. follicles ≥ 14 mm	.355^**^
Progesterone-to-follicle index	.584^**^

** Correlation is statistically significant (bilateral).

**Figure 2:**
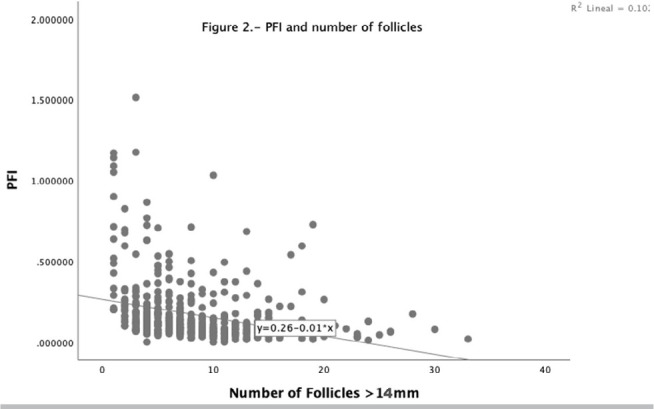
PFI and number of follicles.

Since the progesterone-to-follicle index negatively correlated with the number of follicles, we performed a simple linear regression model. It was corroborated that a higher number of follicles was associated with lower PFI and vice versa (Figure 2).

**Figure 3:**
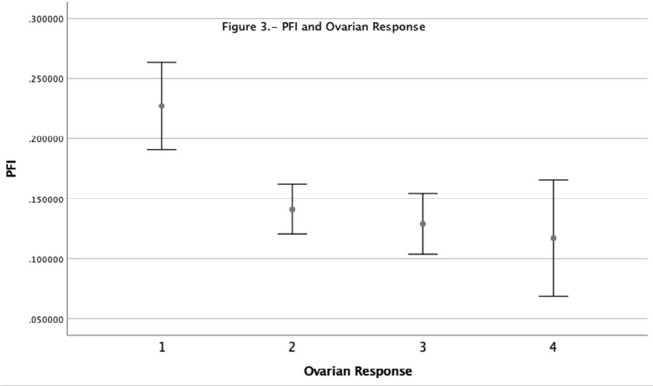
PFI and ovarian responders.

After grouping patients according to their ovarian response, using Bologna Criteria to define the low ovarian responders. We observed that poor ovarian responders have a significantly higher progesterone-to-follicle index than suboptimal, normal, and high responders. These differences among ovarian responses are shown in Figure 3.

Furthermore, employing a Principal Component Analysis, using an eigenvalue of 1, we detected three components representing 59.3% of the accumulated variance. It is vital to notice that component 2 had the highest progesterone level among the three components, a relatively low number of follicles, a low ovarian response, the lowest estradiol level, and the highest progesterone-to-follicle index, as illustrated in Table 3.

**Table 3:** Principal Component Analysis.

Parameter	Component 1	Component 2	Component 3
Age	-0.300	0.184	0.466
BMI	-0.083	-0.253	0.419
Stimulation length	0.204	0.560	0.382
FSH Dosage	0.045	0.482	0.680
Num. follicles ≥ 14 mm	0.880	-0.139	0.043
Progesterone	0.392	0.698	-0.373
Estradiol	0.621	0.056	0.270
Num. Oocytes retrieved	0.833	-0.141	-0.083
PFI	-0.214	0.792	-0.356

Extraction method: Principal Component Analysis.

## DISCUSSION

Our results show that although ovarian response determines progesterone levels, other intrinsic follicular features determine the progesterone levels on hCG day. In addition, the PFI is the ovarian stimulation parameter with the best correlation with progesterone levels on hCG day. After applying the simple linear regression model, we confirmed that PFI has the best predictive capacity among the other parameters. The PCA analysis also showed that the group with fewer retrieved oocytes (patients in component 2) had higher gonadotropin dosage, lowest estradiol production, and the most elevated progesterone per follicle. Our study supports “gate-keeper” mechanisms that prevent premature luteinization from being disrupted in low ovarian responders. In summary, we noticed that patients with low ovarian response have limited capacity to aromatize estradiol, limiting its ability to overcome excessive progesterone levels, leading to an increased progesterone-estradiol ratio.

These findings are in accordance with Wu *et al*. (2015), since they found that in granulosa-cells collected from follicular fluid, FSH receptor (FSHR), aromatase (CYP19A1) and 17-beta-hydroxysteroid dehydrogenase (HSD17B) expression were found down-regulated with advancing age. In contrast, the L.H. receptor and progesterone receptor were up-regulated, thus limiting the capacity of the follicle to produce estradiol and overcoming its capacity to handle progesterone.

In humans, other researchers showed similar results (Fanchin *et al*., 1997; Ou *et al*., 2008; Elgindy, 2011; Shalom-Paz *et al*., 2015; Luborsky *et al*., 2002). Other authors showed that patients with lower ovarian responses had lower estradiol and higher progesterone levels and FSH consumption compared to regular responders. So increased progesterone/estradiol ratio unrelated to preovulatory LH elevation and irrespective of LH/hCG content of gonadotrophin could be associated with poor ovarian response and dysmature follicles (Fanchin *et al*., 1997; Ou *et al*., 2008). These findings could explain the cause of a lower cumulative pregnancy rate in low ovarian responders, since they do not have an increased number of oocytes and embryos compared to high ovarian and regular ovarian responders. The optimal late follicular progesterone /estradiol ratio threshold could be between 0.45 to 0.55 (Elgindy, 2011; Shalom-Paz *et al*., 2015). However, some authors have suggested that cutoff values such as >1 are associated with poorer clinical outcomes (Fanchin *et al*., 1997). Since elevated progesterone on hCG day does not severely affect IVF results in regular and high responders, it is reasonable to explore other mechanisms by which low ovarian responders develop a premature progesterone rise. Previous reports have shown that low ovarian responders exhibit a trend toward accelerated luteinization (Luborsky *et al*., 2002). Similar observations were published by Karl *et al*. (2021). They observed that high FSH doses in heifers with small ovarian reserves reduced the capacity of ovulatory follicles to produce estradiol, ovulation and form a corpus luteum (Karl *et al*., 2021).

The main strength of our study is its data analysis strategy to present novel evidence that low ovarian reserve seems to be an etiology of P elevation in ART cycles, employing GnRH antagonist protocols. In contrast, our study does have several limitations. First, the retrospective nature of our study, the fact that it was performed at a single institution in Mexico City, and the relatively small sample size. Thus, our findings need to be reproduced in larger patient series with longer follow-up and evaluation of live-birth rate.

Future randomized controlled trials (RCTs) evaluating the effect of premature luteinization of granulosa cells in low ovarian responders having live birth rate as the main outcome are encouraged. Ideally, the study design should control the effects of main confounders (age, smoking status, gonadotrophin dosage, and characteristics of the embryos transferred). It would also be interesting to assess the effect of premature progesterone rise prior to the first frozen-thawed embryo transfer cycle in low ovarian responders, as well as cost-effectiveness studies.

## CONCLUSIONS

Poor ovarian responders produce more progesterone per follicle, suggesting the possibility of an altered gate-keeper mechanisms that prevent premature luteinization. More prospective trials are needed to validate these results.
